# Metagenomic analysis of mother-infant gut microbiome reveals global distinct and shared microbial signatures

**DOI:** 10.1080/19490976.2021.1911571

**Published:** 2021-05-07

**Authors:** Shaopu Wang, Shuqin Zeng, Muireann Egan, Paul Cherry, Conall Strain, Emilene Morais, Patrick Boyaval, C. Anthony Ryan, Eugene Dempsey, R. Paul Ross, Catherine Stanton

**Affiliations:** a APC Microbiome Ireland, University College Cork, Cork, Ireland; b Food Biosciences Department, Teagasc Food Research Centre, Moorepark, Fermoy, Co. Cork, Ireland; c DuPont Nutrition and Biosciences, Paris, France; d Department of Paediatrics and Child Health, University College Cork, Cork, Ireland; e INFANT Centre, University College Cork, Cork, Ireland

**Keywords:** Mother, infant, neonate, metagenomics, microbiome, gut, vertical transmission, prediction

## Abstract

Emerging evidence indicates maternal microbiota as one major reservoir for pioneering microbes in infants. However, the global distinct and identical features of mother–infant gut microbiota at various taxonomic resolutions and metabolic functions across cohorts and potential of infant microbial prediction based on their paired mother’s gut microbiota remain unclear. Here, we analyzed 376 mother–infant dyads (468 mother and 1024 infant samples) of eight studies from six countries and observed higher diversity at species and strain levels in maternal gut microbiota but not their metabolic functions. A number of 290 species were shared in at least one mother–infant dyad, with 26 species (five at strain level) observed across cohorts. The profile of mother–infant shared species and strains was further influenced by delivery mode and feeding regimen. The mother-sourced species in infants exhibited similar strain heterogeneity but more metabolic functions compared to other-sourced species, suggesting the comparable stability and fitness of shared and non-shared species and the potential role of shared species in the early gut microbial community, respectively. Predictive models showed moderate performance accuracy for shared species and strains occurrences in infants. These generalized mother–infant shared species and strains may be considered as the primary targets for future work toward infant microbiome development and probiotics exploration.

## Introduction

The human gut microbiome has been increasingly revealed to be involved in the health and development of the host, and changes in the microbiota are linked to various chronic diseases (e.g., allergic diseases, obesity, and diabetes).^1–3^ This link has led to considerable attention toward exploring the gut microbiota as a potential novel target for disease prevention and/or treatment. The major establishment of the microbiota and its symbiosis with the host begin during and after birth and are influenced by various intrinsic and extrinsic factors, including birth mode and feeding regimen in early life.^4–7^

The maternal gut microbiota is one of the main microbial reservoirs in humans and a source of  continual exposure to infants in early life. This serves as a source of  pioneering microbes and colonizers  in the offspring to shape the long-term succession of the gut microbial community.^4,8^ Although the early-life microbiota has been substantially described recently,^4,5,9–11^ thus far, how well distinct and shared features of microbiota between mothers and infants, from taxonomic profiles, structural variants among strains, to metabolic functions have not been exhaustively compared across multiple high-resolution metagenomic cohorts. The developmental trajectory of the gut microbial ecosystem in nature is also highly dynamic in early life and differs widely among individuals.^10^ Large cohorts with longitudinal sampling from matched mother–infant dyads are necessary to decrease bias arising from individual heterogeneity and uncover successive microbiota similarities and differences within or between mothers and infants. The generalized results and the consensus mother–infant shared microbial species and strains across cohorts can deepen our understanding of the maternal contribution to the development of infant gut microbiota and unique characterization of microbiota from mothers and infants.

Furthermore, the potential to predict the gut microbes in infants is not known due to the complexity of the microbial community and interactions (e.g., cross-feeding) to one another.^12^ Given the frequent mother–infant contact in early life thus inducing exchanges of microbiota between mother and infant,^13,14^ we speculated that occurrences of the specific microbes in infants that are shared with their mothers may be predicted based on the maternal gut microbiota but this area is still unexplored. The promising outcomes can provide an early estimation of the gut microbiota patterns in infants (e.g., missing microbes in infants), thus informing us to design potential microbial intervention strategies, such as specific probiotic supplementation for mothers and infants.^14^

To address these questions, we presented a large-scale meta-analysis of eight publicly available shotgun metagenomic-based studies with longitudinal sampling of 376 mother–infant dyads, including 468 maternal fecal samples ranging from pregnancy, delivery to postpartum, and 1024 infant fecal samples from birth up to one year of age.^8,9,15–20^ This enlarged data set enabled us to comprehensively investigate (i) the distinct aspects in gut microbial composition, strain heterogeneity, and metabolic functions between mothers and infants; (ii) the overall and core panel of microbial mother–infant shared biomarkers across cohorts; (iii) the microbial mother-to-infant vertical transmission at strain level; (iv) influences of maternal characteristics on the infant gut microbiota; (v) alterations of shared species and strains by clinical confounders, especially paying attention to the mode of delivery and feeding regimen in early life; and more importantly (vi) prediction of mother–infant shared species and strains occurrences in infants with different machine learning algorithms based on maternal gut microbiota.

## Results

### Meta-analysis of publicly available shotgun metagenomic sequencing data sets

The justification of studies to be included or excluded are summarized in Materials and methods and Supplementary Table S1. Eight studies that shotgun-sequenced fecal samples from infants and paired mothers were selected in this meta-analysis to identify and characterize the distinct and shared features of microbiota between mothers and infants including microbial taxa at strain level and metabolic pathways. These eight metagenomic studies were conducted in six countries, including two from Italy,^15,17^ one from Luxembourg,^20^ three from Northern Europe (one from Sweden,^9^ two from Finland^8,18^), one from the United States,^16^ and one from the United Kingdom^19^ (Table 1). After removing non-stool samples if present and subjects missing either mother or infant samples from each study, a total of 376 matched mother–infant pairs were included for subsequent analysis, comprising samples from birth up to 12 months of age for infants (*n* = 1024), and maternal samples from pregnancy, delivery, and postpartum (*n* = 468). A summary of the metadata of included studies is presented in Supplementary Table S1. The metagenomic shotgun sequencing data of all the included studies were reprocessed using consistent bioinformatic analyses for taxonomy profiling, namely, MetaPhlAn2^58^ and StrainPhlAn^21^ for species and strains, respectively, and HUMAnN2^60^ for functional profiling (Materials and methods).

**Table 1.** Number of paired mother–infants from included studies

**References**	**Country**	**No. of paired mothers and infants**	**No. of maternal samples**	**No. of infant samples**	**Mean inital paired reads (million)** **95% confidence interval (95% CI)**
Bäckhed et al.^9^	Sweden	98	98	294	39.9, 95% CI = [39.1; 40.7]
Chu et al.^16^	United States	10	15	12	28.0, 95% CI = [19.9; 36.0]
Asnicar et al.^15^	Italy	5	8	8	27.8, 95% CI = [23.1; 32.6]
Ferretti et al.^17^	Italy	21	21	81	35.5, 95% CI = [26.9; 44.0]
Pärnänen et al.^18^	Finland	16	32	32	12.5, 95% CI = [11.7; 13.2]
Yassour et al.^8^	Finland	38	106	152	33.2, 95% CI = [31.4; 34.9]
Wampach et al.^20^	Luxembourg	13 (one twin birth)	13	35	32.7, 95% CI = [27.7; 37.7]
Shao et al.^19^	United Kingdom	175 (three twin births)	175	410	19.9, 95% CI = [19.4; 20.4]

## A complete view of distinct gut microbial profiles between mothers and their infants

**Figure 1.**
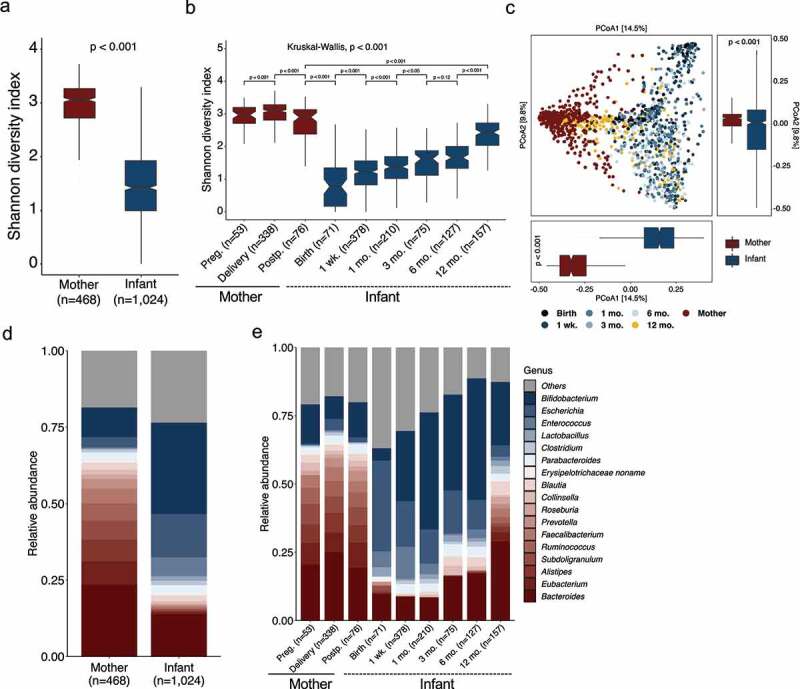
**Distinct diversity and composition of gut microbiota from mother–infant dyads.** (a) Species alpha diversity (Shannon diversity index) of mothers (*n*=468) and infants (*n*=1024). The *p*-value was computed using a blocked (by “study”) Wilcoxon test from R package “coin”. (b) Species alpha diversity (Shannon diversity index) of stools from mothers and infants stratified by “sampling time points” into three categories for mothers and six categories for infants. The *p*-values were computed using Wilcoxon test. The overall *p*-value in (b) (on top) was calculated with a blocked (by “study”) Kruskal–Wallis test from R package “coin”. (c) Principal coordinate analysis (PCoA) of samples of mothers and infants from all the eight included studies based on Bray–Curtis dissimilarity of species. The boxplots on the right side and below show samples of mothers and infants projected onto the first two principal coordinates, respectively. The *p*-values were calculated by the adonis (permutations=1000) function from the R package “vegan” for the PCoA plot, and by a blocked (by “study”) Wilcoxon test from R package “coin” for the boxplots. (d, e) Comparisons of the mean relative abundance of gut genera from mothers and infants (d), and stratified by “sampling time points” (e). Only the genera that differed (FDR < 0.05, Wilcoxon test blocked by “study”) between mothers and infants, with > 0.3% mean relative abundance and at least 5% prevalence among maternal and infant samples across all “sampling time points”, respectively, are plotted. The mean relative abundance of genera with blue are higher in stools of infants, and genera with red are higher in mothers

Comparing the microbial relative abundance between mothers and infants across the eight included studies revealed distinct microbial communities in both diversity and composition (Figure 1; Supplementary Figure S1). Consistently higher richness (Wilcoxon rank-sum test with blocked by “study” (hereafter referred to as blocked Wilcoxon test), *p* < .001) and alpha diversity (Shannon diversity index and Simpson diversity index; blocked Wilcoxon test, *p* < .001) were observed in mothers (Figure 1a; Supplementary Figure S1a). These differences declined as infants grew toward an adult-like gut microbiota until one year of age (Figure 1b; Supplementary Figure S1b), which was reflected by stratification of longitudinal maternal and infant samples based on the “sampling time points” (Materials and methods). Principal coordinate analysis (PCoA) ordination of Bray–Curtis dissimilarity that clustered maternal and infant samples demonstrated a mother–infant differentiation, but an increasing convergence of gut microbiota between mothers and their infants (adonis PERMANOVA test, *p* < .001, permutations = 1000) (Figure 1c). In maternal fecal samples, Bacteroidetes (40.3%) and Firmicutes (38.9%) dominated the microbial community, while infants within one year of age had less Bacteroidetes [17.9%; blocked Wilcoxon test, false discovery rate (FDR) < 0.001] and Firmicutes (26.4%; blocked Wilcoxon test, FDR < 0.001) but a higher abundance of Actinobacteria (32.5% vs. 12.1% for mothers; blocked Wilcoxon test, FDR < 0.001) and Proteobacteria (21.7% vs. 5.0%; blocked Wilcoxon test, FDR < 0.001) (a full list of significant taxa in Supplementary Table S2; Supplementary Figure S1c). The mean relative abundance of 113 (of 282) genera and 352 (of 814) species differed between mothers and infants (blocked Wilcoxon test, FDR < 0.05; Supplementary Table S2). Of the differed genera with prevalence of at least 5% and mean relative abundance > 0.3%, *Bifidobacterium, Escherichia, Enterococcus, Lactobacillus, Clostridium*, and *Parabacteroides* were more enriched in stools of infants than in mothers. Genera including *Bacteroides, Eubacterium, Alistipes, Subdoligranulum*, and *Ruminococcus* dominated in mothers (Figure 1d). A total of 16 (of 42) species with mean relative abundance (>0.1%) and high prevalence (at least 5%) were higher in stools from infants than mothers (Supplementary Figure S1d). Among these, three species were from *Bacteroides* (*Bacteroides fragilis, Bacteroides dorei*, and *Bacteroides faecis*), and four species were from *Bifidobacterium* (*Bifidobacterium longum, Bifidobacterium bifidum, Bifidobacterium pseudocatenulatum*, and *Bifidobacterium catenulatum*) (Supplementary Figure S1d). Similar to microbial diversity, an accelerated change was observed between the age of 6–12 months, when an increase in *Bacteroides* but a decrease in *Bifidobacterium* was observed (Figure 1e; Supplementary Table S3).

## Meta-analysis of gut microbial taxa shared by mothers and their infants

**Figure 2.**
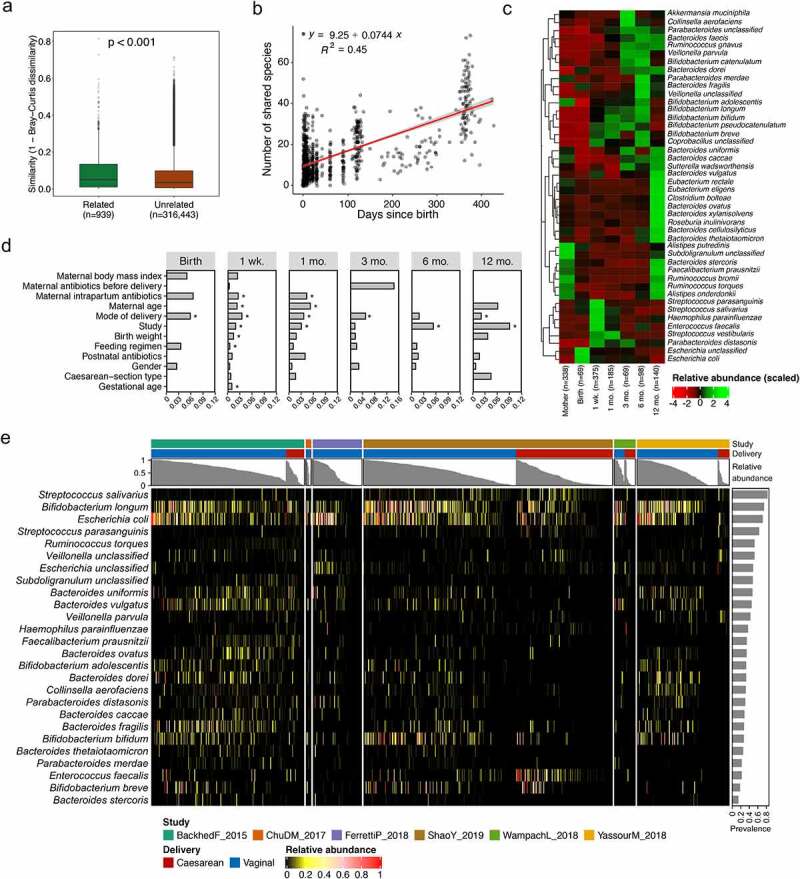
**Feature of microbiota shared by mother–infant dyads**. (a) Similarity of species-level composition profiles between related and unrelated mother–infant dyads as measured by Bray–Curtis dissimilarity. (b) Number of mother–infant shared species increased as infants aged, with regression line (red). (c) Longitudinal changes in the relative abundance of mother–infant shared species, with > 0.1% mean relative abundance and at least 5% prevalence of infant samples across all “sampling time points”. (d) Horizontal bars indicated the proportion of variance (*R*^2^) explained by clinical covariates stratified by “sampling time points” that are associated with mother–infant shared species in the model as determined by PERMANOVA. Asterisk denotes the significance (FDR < 0.05) of each covariate as determined by PERMANOVA. (e) Abundance of a core set of 26 mother–infant shared species across six studies investigated, with average relative abundance > 0.1% and at least 5% prevalence among all the infant samples. The column annotations on the top indicate the study, mode of delivery and the total relative abundance of 26 shared species for each sample. Bar plot on right side indicated the prevalence of each core shared species among all mother–infant dyads (*n* = 342, including four twin births)

Given evidence indicating the vertical transmission of maternal microbes to infants and the early and frequent interactions between mothers and infants, we next sought to determine members of the gut microbiota that are shared by infants and their paired mothers across cohorts. Compared to maternal samples collected at delivery, only five more species (*Butyricimonas synergistica, Leuconostoc mesenteroides, Leuconostoc pseudomesenteroides, Peptostreptococcus anaerobius*, and *Clostridium methylpentosum*) were further identified to be shared by mother–infant dyads from the postpartum period (sampling after seven days of infant age). We thus focused on the maternal microbiota present at delivery (sampling from birth to seven days of infant age) from a subset of six studies (Bäckhed et al.,^9^ Chu et al.,^16^ Ferretti et al.,^17^ Shao et al.,^19^ Wampach et al.,^20^ and Yassour et al.^8^) with collecting maternal samples at delivery, resulting in 342 mother–infant dyads with four twin births. We firstly compared the similarity between infants with their own mothers or unrelated mothers across the six studies. The microbiota within related mother–infant pairs displayed significantly (Wilcoxon test, *p* < .001) higher similarity compared to unrelated pairs (Figure 2a). Within related mother–infant pairs, a total of 290 shared species between mothers and infants in at least one mother–infant pair were identified regardless of the relative abundance (Supplementary Table S4), and the number of shared species increased as infants aged (Figure 2b). Expanding on the findings by Ferretti et al.^17^ that shared species were notably lower in relative abundance in mothers than in infants, we found that this pattern was species- and age-dependent. Shared species of *Escherichia* (*Escherichia coli), Streptococcus* (*Streptococcus salivarius* and *Streptococcus parasanguinis), Enterococcus* (*Enterococcus faecalis*), and *Haemophilus* (*Haemophilus parainfluenzae*) were especially present in higher abundance in neonates in the first week of life; conversely, shared species of *Collinsella* (*Collinsella aerofaciens), Klebsiella, Bifidobacterium* (*B. catenulatum, Bifidobacterium adolescentis, B. longum, B. bifidum, B. pseudocatenulatum*, and *Bifidobacterium breve), Veillonella* (*Veillonella parvula*), and *Parabacterioides* (*Parabacterioides merdae*) were typically enriched in infants from one to six months (Figure 2c; Supplementary Figure S2a). In addition, shared species of *B. fragilis* and *Akkermansia muciniphila* were enriched in infants from one to six months of life. At one year of age, the shared species between infants and their mothers had an almost similar relative abundance, apart from the genera of *Lactobacillus, Clostridium* (*Clostridium bolteae), Blautia, Bacteroides* (e.g., *Bacteroides thetaiotaomicron, Bacteroides cellulosilyticus, Bacteroides xylanisolvens, Bacteroides ovatus*, and *Bacteroides vulgatus*), and *Roseburia* (*Roseburia inulinivorans*) that were present with higher relative abundances in infants (Supplementary Figure S2a for genus; Figure 2c for species).

Influences of clinical covariates on the microbiota acquisition and progression in early life have been frequently documented;^11,19^ however, whether these effects vary dependant on the source of the microbiota, in particular influences on those shared with mothers, remains unclear. We thus quantified the influence of 12 covariates available in studies (Materials and methods) stratified by “sampling time points” on the overall composition of mother–infant shared microbiota at the species level. The majority of clinical covariates influenced the relative abundance of shared species at the first week of life (Figure 2d; PERMANOVA, FDR < 0.05). The effect of mode of delivery on the profile of the shared microbial community decreased gradually in the first year of life, and as infants aged, the covariate of “study” gradually dominated the contribution of variation in the shared species composition (Figure 2d; PERMANOVA, FDR < 0.05), which is in line with the TEDDY cohort where the geographical location was associated with the development of the entire microbiota in infants.^3^

## A panel of gut microbial mother–infant shared biomarkers reproducible across cohorts

Among the mother–infant shared species, 43 species were identified with >0.1% mean relative abundance and at least 5% prevalence across all infant samples. Furthermore, 26 shared species (23 classified and three unclassified) were present in at least one mother–infant pair within each of six studies (Figure 2e; Supplementary Figure S3a), thus referred to as a core set of shared species in the current study, belonging to 12 genera that were dominated by *Bacteroides* (eight species) and *Bifidobacterium* (four species). The core set of shared species consisted of 3.6% of the overall infant microbial species pool (26 of 714 species), however, these species represented high abundance with more than 50% of total microbiome in 56% of investigated infant samples (Supplementary Figure 2e). We further found that the prevalence of core shared species differed, with *S. salivarius* (281 of 342 dyads) as the most prevalent species followed by *B. longum* (253 of 342 dyads) and *E. coli* (242 of 342 dyads). *Bacteroides stercoris* was shared in a limited number of 47 dyads (Figure 2e). Considering the potential influence from Cesarean section (C-section) birth mode, we excluded any infants born by C-section and their paired mothers from the data set. This resulted in the removal of *E. faecalis, Faecalibacterium prausnitzii, V. parvula*, and *Subdoligranulum* spp. from the set of 26 shared microbes. When exploring a subset of infants born by C-section, we found that the shared species were study-dependent, where only two species (*B. longum* and *E. coli*) were shared in five of the six studies with C-section-born infants, while the infants born vaginally shared 22 species with their mothers across all six studies (Supplementary Figure S3b and Figure S3c).

## Mother gut microbial species show high strain heterogeneity compared with those of infants

**Figure 3.**
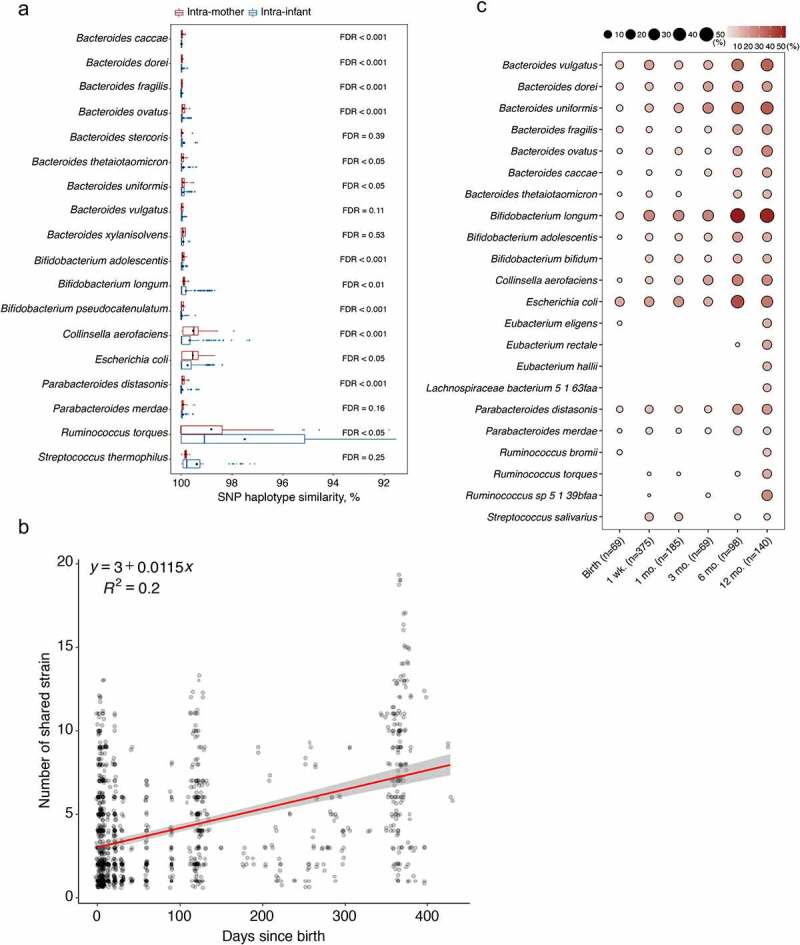
**Strain-level analysis of the mother-to-infant gut transmitted species**. (a) SNP haplotype similarity of each species based on all pairwise comparisons (dominant strain per species) of the marker genes, and stratified to intra-mother and intra-infant comparisons. Species containing at least 10 comparisons in both strata are shown. The significance (*p*-values adjusted by Benjamini–Hochberg FDR method on the right of the bars) of the difference in similarity between mothers and infants was determined by Wilcoxon test. The solid black point indicates the mean of SNP haplotype similarity. (b) Number of shared strains increased as infants aged indicating by the regression line (red). (c) Dynamic prevalence of mother–infant shared strains stratified by “sampling time points”. Only the frequently mother–infant shared strains present in at least 10% of samples (indicated in the bracket) in at least one period are shown

The gut microbiota is highly malleable to numerous extrinsic and intrinsic factors, such as diet, medication, and host immune system.^22,23^ Comparisons between the mother and infant microbiota are typically limited to genus and species levels and have not yet been explored to strain level. Based on the current consensus of the definition of a microbial strain,^21^ we took advantage of shotgun metagenomic sequencing data to perform strain-level analysis by calling a single-nucleotide polymorphism (SNP) on conserved and unique species-specific marker genes using StrainPhlAn for all the detected species from MetaPhlAn2. The produced marker gene-based SNP haplotype of the dominant strain per species was referred to as SNP haplotype.^24^ The longitudinal samplings from mothers and their infants in a subset of studies (Asnicar et al.,^15^ Chu et al.,^16^ Pärnänen et al.,^18^ and Yassour et al.^8^) allowed us to compare the strain heterogeneity within species longitudinally between intra-mother and intra-infant (Figure 3a) by comparing the sequence of SNP haplotype. We found that strain similarity within species sampling at different time points from infants was consistently higher than in mothers (Wilcoxon test, FDR < 0.05), except *for B. thetaiotaomicron, B. longum*, and *Ruminococcus torques*. Species of *R. torques, E. coli*, and *C. aerofaciens* showed a wide range of strain diversity in mothers and infants, but strains shared average marker genes similarity of 98.98%, 95% confidence interval [95% CI] = [98.80%; 99.16%] in SNP haplotype comparisons. The majority of the investigated species belonging to genera of *Bacteroides* and *Bifidobacterium* were characterized with very high strain similarity within the subjects, with respective marker genes similarity averages of 99.90%, 95% CI = [99.89%; 99.92%] and 99.67%, 95% CI = [99.61%; 99.73%] in intra-infant comparisons, and 99.87%, 95% CI = [99.86%; 99.89%] and 99.80%, 95% CI = [99.76%; 99.83%] in intra-mother comparisons.

## Characterization of gut microbial mother-to-infant vertical transmission at strain level

To provide strong evidence of microbial mother-to-infant vertical transmission, the metagenomic sequencing data were further explored to identify the mother–infant shared species at strain level. A total of 103 strains were shared by mother–infant dyads of the six studies where maternal samples at delivery were available (Bäckhed et al.,^9^ Chu et al.,^16^ Ferretti et al.,^17^ Shao et al.,^19^ Wampach et al.,^20^ and Yassour et al.^8^). This accounted for 35.5% (103/290) of the total shared species. A total of five strains (*Bacteroides uniformis, B. vulgatus, B. longum, Parabacteroides distasonis*, and *P. merdae*) that were part of the set of 26 species were confirmed in at least one mother–infant pair in each of the six studies. Similar to the pattern of shared species, the number of shared strains increased gradually as infants aged, from 2.93 strains on average per subject (95% CI = [1.95; 3.91]) in neonates at birth to 6.59 strains (95% CI = [5.86; 7.32]) in infants at one year of age (Figure 3b). Of these shared strains present in at least 10% of samples in each corresponding period, strains from the genus *Bacteroides* were the most prevalent and persistent colonizers in early life, followed by *Bifidobacterium* (Figure 3c). At birth, *E. coli* (17.4%), *B. vulgatus* (11.6%), and *B. longum* (10.1%) were the most frequently found strains in the neonatal gut. As infants aged in the first year of life, the frequency of *B. longum* strains increased, followed by *B. uniformis, B. vulgatus, E. coli*, and *B. ovatus* strains (Figure 3c; see the complete dynamic prevalence of all mother–infant shared strains in Supplementary Table S5).

Apart from the maternal gut as one of the most likely sources of microbiota for infants due to the comparable microbial ecosystem (referred to here as shared species), the source of the early colonizers is multiple, including mother’s oral cavity and skin, and surrounding environment^17,25^ (referred to here as non-shared species). By applying an SNP calling approach on the marker genes from StrainPhlAn, we calculated the strain similarity by comparing the sequence of SNP haplotype within shared or non-shared species from the longitudinal infant samples. The result indicated that each group of species had high strain similarity, and no significant differences except *B. dorei* and *Streptococcus vestibularis* were found between shared and non-shared species (Wilcoxon test, FDR > 0.05; Supplementary Figure S2b). However, these non-shared strains exhibited reduced abundance in metabolic pathways (Supplementary Figure S4), in particular for proteinogenic amino acid biosynthesis, purine nucleotide biosynthesis, vitamin biosynthesis, cell wall biosynthesis, and coenzyme-A-biosynthesis.

## High overlap in gut microbial functional capacities between mothers and infants

**Figure 4.**
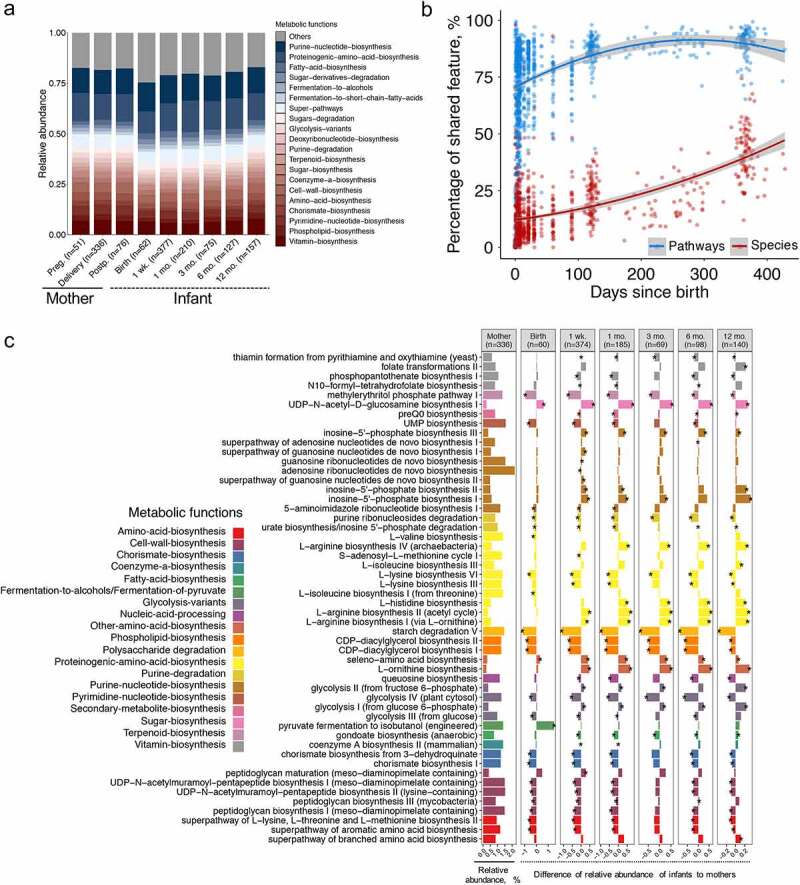
**Gut microbiota functional capacity shared by infants with their mothers**. (a) Dynamic changes of microbial metabolic functions that differed (FDR < 0.05, Wilcoxon test blocked by “study”) between mothers and infants, with > 1% mean relative abundance and at least 5% prevalence among maternal and infant samples across all “sampling time points”, respectively. The mean relative abundances of metabolic functions in blue are higher in infants, and metabolic functions in red are higher in mothers. (b) Infants and mothers shared, on average, four times of number of metabolic pathways (76.6%) than species (16.8%) (Wilcoxon test blocked by “study, *p* < .001). The curves show quadratic fit for the percentage of shared features with infants age, and shaded area shows 95% confidence interval for each fit. (c) Shared metabolic pathways by mothers and infants stratified by “sampling time points”, with significant difference (Wilcoxon test blocked by “study”, FDR < 0.05, bar with asterisk) between mothers and infants at least one time point. Metabolic pathways (> 0.5% mean relative abundance and at least 5% prevalence in infant samples across all “sampling time points”) are colored based on the metabolic functions

Accompanying our taxonomic profiling, a direct analysis of the microbial functional capacities in terms of comparisons between mothers and infants is possible with shotgun metagenomic data in our meta-analysis. This provides deep insights into the functional transmission and maturation of microbes in early life. A total of 518 metabolic pathways involving 111 functions (as described by MetaCyc metabolic pathway hierarchy classification) were identified across all the included maternal and infant fecal samples. Surprisingly, the alpha diversity of pooled samples from mothers and infants were comparable except for functional richness (Supplementary Figure S5a). Further, PCoA indicated that metabolic pathways among infants were more variable than that of mothers (Supplementary Figure S5b; adonis PERMANOVA test, *p* < .001, permutations = 1000). Among these 518 metabolic pathways, 361 resulting in 86 metabolic functions were different (blocked Wilcoxon test, FDR < 0.05) between mothers and infants (Supplementary Table S6). Infants aged from birth up to 1 year of age had a lower abundance of metabolic functions, such as vitamin and phospholipid biosynthesis, but had a higher abundance of functions such as biosynthesis of purine nucleotides, proteinogenic amino acids, fatty acids, and fermentation to short-chain fatty acids compared to mothers (Figure 4a; Supplementary Figure S5c).

We further analyzed the shared functional pathways by mothers and infants across ages, and it showed mothers and infants shared, on average, four times the number of metabolic pathways (76.6%, 95% CI = [75.5%; 77.7%]) than species (16.8%, 95% CI = [15.9%; 17.7%], blocked Wilcoxon test, *p* < .001) (Figure 4b), consistent with previous studies.^15,26^ A total of 405 out of 518 metabolic pathways were found to be shared in at least one mother–infant dyad. Among these 405 metabolic pathways, 282 pathways in mothers were different from infants in at least one time point (blocked Wilcoxon test, FDR < 0.05; Supplementary Table S7), showing changes in the first year of life. The majority of shared metabolic microbial metabolic pathways that differed between mothers and infants belonged to purine nucleotide biosynthesis and proteinogenic amino acid biosynthesis (Figure 4c). Among pathways of proteinogenic amino acid biosynthesis, the biosynthesis of L-arginine (I, II, and IV) in neonatal fecal microbiota was comparable to maternal microbiota at birth. The relative abundance of L-arginine biosynthesis pathway increased continuously until one year of age, resulting in higher abundance than that of mothers, which was opposite to the case of biosynthesis of L-lysine (III, VI). In addition, infants were rich in sugar nucleotide biosynthesis pathway (UDP-N-acetyl-D-galactosamine biosynthesis I). Nevertheless, the infant microbiota showed a higher potential for folate biosynthesis while displaying a lower potential for starch degradation, cell wall biosynthesis, and phospholipid biosynthesis, consistent with previous observations.^15,27^ The increased abundance of starch degradation pathway was observed as infants aged, indicating that the early microbiota is adapted to the introduction and metabolism of plant-derived glycans.

## Influences of maternal characteristics on the infant gut microbiota

We extracted the maternal characteristics [i.e., maternal body mass index (BMI) before pregnancy, maternal antibiotics before delivery, maternal intrapartum antibiotics, and maternal age] from a subset of the eight included studies, depending on the availability of the investigated characteristics, and examined their effects on the gut microbiota of the infant (Supplementary Table S1). The result showed that the richness of gut microbiota from infants born to overweight or obese (OWOB) mothers was decreased (Wilcoxon test, *p* < .05) compared to those born to normal BMI mothers (Supplementary Figure S6a). The same pattern with lower alpha diversity (Wilcoxon test, *p* < .05; richness and Shannon diversity index) was also observed from infants born to mothers receiving antibiotics during delivery (Supplementary Figure S6c). Our MaAsLin analysis indicated that *Bacteroides* (*B. vulgatus*) and *Parabacteroides* (*P. merdae*) were higher (MaAsLin, FDR < 0.25) and *Citrobacter* and *Enterococcus* (*E. faecalis*) were lower in infants born to OWOB mothers (Supplementary Table S8). In addition, exposing mothers to antibiotics during delivery or mothers aged more than 30 years decreased the abundance of *Bifidobacterium* but increased *Enterobacter cloacae* in the infant gut microbiota. There was no difference found for the alpha diversity of the infant gut microbiota born to mothers with or without antibiotics during pregnancy but the increased abundance of genera *Bilophila* and *Megasphaera* resulted from maternal antibiotic exposure during pregnancy (Supplementary Figure S6b; Supplementary Table S8).

## Influences of mode of delivery and breastfeeding on the infant gut microbiota

**Figure 5.**
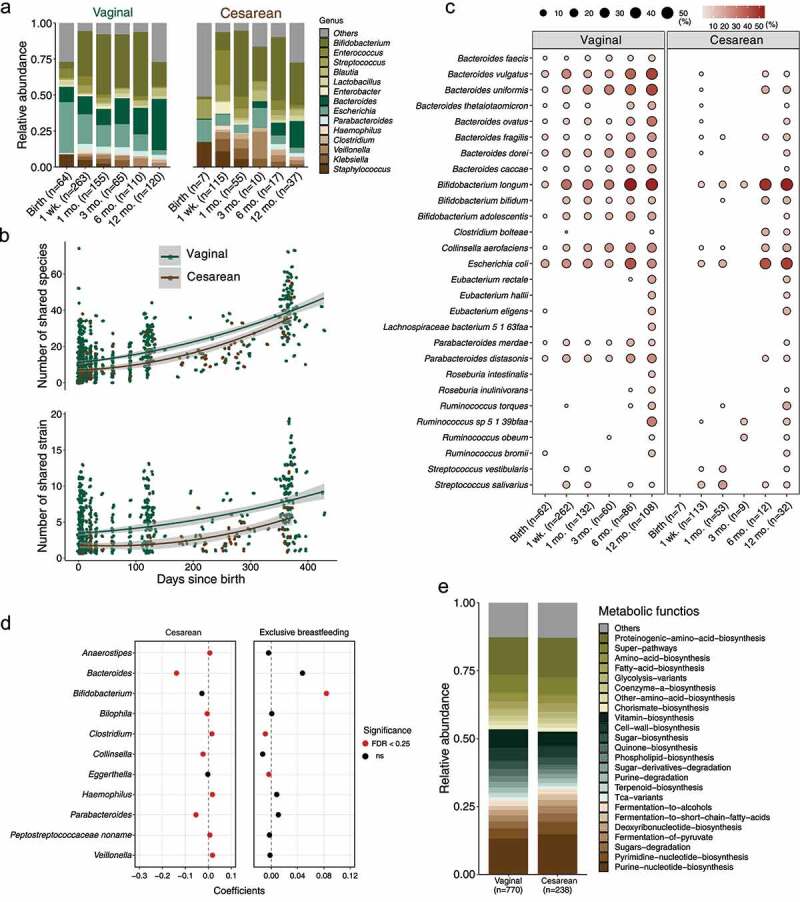
**Influence of mode of delivery and feeding regimen on the mother–infant shared gut species and strains**. (a) Longitudinal changes in the mean relative abundance of genera of microbiota in infants stratified by “sampling point times” and mode of delivery (Cesarean section vs. vaginal), for genera with > 1% mean relative abundance from infant samples across all “sampling point times”. (b) Longitudinal changes in the number of species and strains shared by mothers and infants stratified by mode of delivery (*p* < .001 for shared species, and *p* < .001 for shared strains, Wilcoxon test blocked by “sampling time points”). (c) Dynamic prevalence (%) of shared strains by mothers and infants stratified by “sampling time points” and mode of delivery. Only the frequently shared strains present in at least 10% of samples (indicated in the bracket) in at least one period of either vaginally- or Cesarean section-born infants are shown. (d) Forest plot illustrated the coefficients of shared genera (MaAsLin, FDR < 0.25 for delivery or feeding regimen) influenced by mode of delivery and feeding regimen (exclusive vs. non-exclusive breastfeeding). (e) Comparisons of metabolic functions between vaginally and Cesarean section-born infants, with > 1% mean relative abundance across all infant samples (MaAsLin, FDR < 0.25). The mean relative abundance of genera of infant samples across all “sampling point times” (a) and metabolic functions (e) with blue are higher (MaAsLin, FDR < 0.25) in vaginally born infants; genera/metabolic functions with red are higher (MaAsLin, FDR < 0.25) in infants via Cesarean section; and no differences (MaAsLin, FDR > 0.25) for genera/metabolic functions with yellow

Mode of delivery is recognized as an important driver for early gut microbial acquisition and development, in particular for the first year of life.^7,13^ Compared to vaginal delivery, the extent and alteration of gut microbiota resulting from C-section remain controversial, which is due in part to differences in sampling time points, cohort sizes, and sequencing strategies.^16,19,20^ Our meta-analysis with a large combined data set of shotgun metagenomic sequencing data revealed that gut microbiota from vaginally born infants were enriched (MaAsLin, FDR < 0.25) with genera of *Bacteroides, Escherichia*, and *Parabacteroides*, as well as species of *B. longum* and *B. adolescentis* (Figure 5a; Supplementary Table S9) in terms of their mean relative abundance across the first year of life, even when correcting for the infant age at the point of sampling, along with the above-mentioned 12 covariates and sequencing depth. Samples from C-section-born infants instead were dominated (MaAsLin, FDR < 0.25) by species from genera of *Staphylococcus, Klebsiella, Veillonella, Clostridium*, and *Haemophilus*. In addition, the relative abundance of these genera was changed dynamically over the first year of life, namely, the greatest differences in the microbial profile were observed between vaginally- and C-section-born infants within the three months, and afterward gradually decreased in infants in the first year of life. This was also illustrated by a gradual decrease (Bray–Curtis dissimilarity) in the effect of mode of delivery on the overall microbial composition of genera (i.e., *R*^2^ decreasing from 0.075 at one week to 0.043 at one year of age; Supplementary Figure S7). In the meconium, microbial community structure (Bray–Curtis dissimilarity) did not differ significantly (adonis PERMANOVA test, FDR = 0.06, permutations = 1000), consistent with previous studies.^28,29^

In addition, infants born by C-section shared decreased number of species (on average 10.1, 95% CI = [8.88; 11.3]) and strains (2.42, 95% CI = [2.03; 2.80]) than those born vaginally (species: 16.1, 95% CI = [15.1; 17.1], Wilcoxon test blocked by “sampling time points”, *p* < .001; strains: 4.40, 95% CI = [4.12; 4.68], Wilcoxon test blocked by “sampling time points”, *p* < .001; Figure 5b). Although the relatively small sample size in the meconium of C-section-born infants, in a longitudinal view, we observed that the transmission of maternal microbial strains occurred mainly in vaginally born infants (on average 10.1, 95% CI = [8.24; 11.9]) at a higher prevalence (Wilcoxon test blocked by “sampling time points”, *p* < .001) than infants born by C-section (3.43, 95% CI = [2.11; 4.75]) in the first year of life (Figure 5c; see the complete dynamic prevalence of all mother-infant shared strains in Supplementary Table S10), in particular species from *Bacteroides* and *Bifidobacterium*. At one year of age, the incidence of mother-to-infant microbial transmission was becoming more comparable between vaginally- and C-section-born infants although still with significant differences (Wilcoxon test, *p* < .001). Regarding the relative abundance of shared species present in infants, species of *Bacteroides* (*Bacteroides caccae, B. dorei, B. fragilis*, and *B. vulgatus), B. adolescentis, B. longum, C. aerofaciens, E. coli*, and *Parabacteroides* (*P. distasonis* and *P. merdae*) decreased in infants born by C-section, but species of *Clostridium bartlettii, H. parainfluenzae, Rothia mucilaginosa, S. parasanguinis*, and *Veillonella* increased (MaAsLin, FDR < 0.25) (Figure 5d; Supplementary Table S11). The exclusive breastfeeding could alleviate partially the microbial changes associated with C-section birth by increasing the abundance of shared *Bifidobacterium* and decreasing the abundance of shared *Clostridium* in early life (MaAsLin, FDR < 0.25) (Figure 5d).

At the functional level, the important metabolic functions of microbiota that differed between vaginally- and C-section-born infants in the first year of life included biosynthesis of purine and pyrimidine nucleotides, fermentation of pyruvate, and fermentation to short-chain fatty acids (higher in C-section-born infants, MaAsLin, FDR < 0.25), as well as biosynthesis of vitamins, cell wall, sugar, and quinone (higher in vaginally born infants, MaAsLin, FDR < 0.25) (Figure 5e).

## Prediction of occurrences of mother–infant shared species in infants

**Figure 6.**
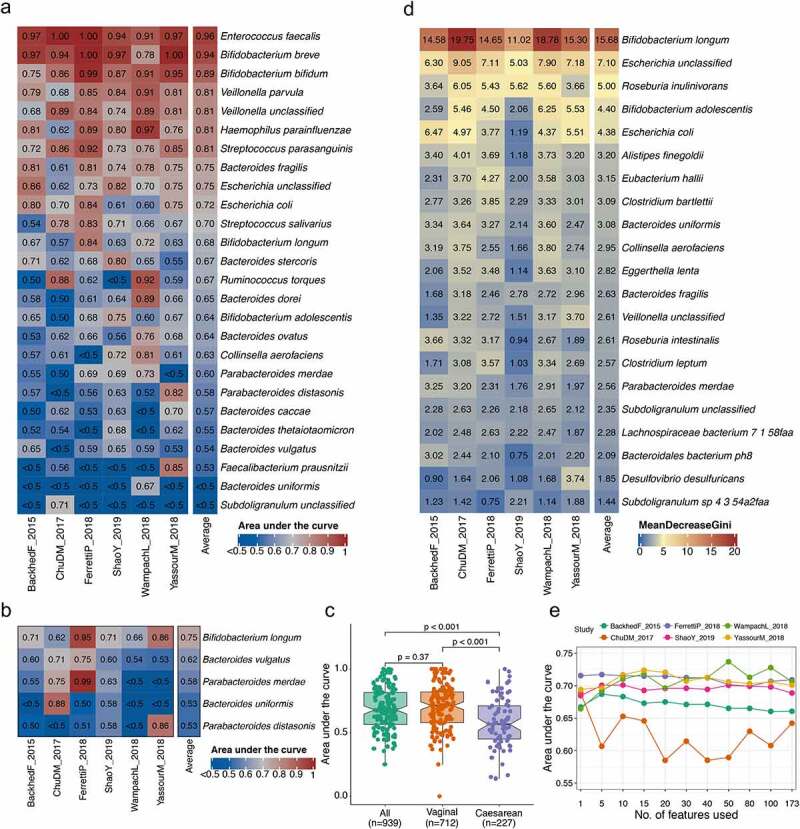
**Assessment of prediction performance of shared species occurrences in infants gut based on maternal gut microbiota**. (a) The area under the curve (AUC) matrix obtained from random forest model with leave-one-study-out (LOSO) approach for the core set of 26 shared species across six studies. Each column refers to the performance of machine learning by taking all but the data set of the corresponding column and applying it to the data set of the corresponding column. (b) The AUC matrix obtained from random forest model with LOSO approach for the five strains across six studies. (c) Changes in the prediction performance with AUC when stratified the samples by mode of delivery. (d) The importance of predictive features reflected by the mean decrease in GINI with LOSO approach for the model of *Bifidobacterium longum*. Only features appearing in the 10 top-ranking features in at least one study are reported. (e) Prediction performances with increasing number of microbial species obtained by retraining the random forest classifier on the top-ranking features identified from the first random forest model training with LOSO approach. The data show the mean of AUC values of the set of 26 species

In order to address the hypothesis whether the maternal gut microbiota could be used to predict the occurrences of species in infants that were shared with their mothers, we performed a leave-one-study-out (LOSO) analysis to provide an unbiased and well-powered assessment for the core set of 26 mother–infant shared species (Materials and methods) with a random forest classifier. We observed the predictive performances in the area under the curve (AUC) score at least 0.70 for 11 species, with the average ranging from 0.96 to 0.53 for 24 species (Figure 6a). The predictive performance was much improved with average AUC values of five repetitions from 0.98 to 0.69 for all species when the pooled samples from six studies were randomly separated into a training set and validation set (70% and 30%, respectively) (Supplementary Figure S8a). The highly comparable performances of prediction (average AUC values from 0.98 to 0.57 for all species) were further confirmed with stochastic gradient-boosting machine learning model (Supplementary Figure S8b; Materials and methods) and LOSO approach. At strain level, we applied the same approach with the random forest and LOSO for five strains that were observed with both transmitted and non-transmitted patterns in each of the six studies to answer the question whether or not the strains inhabiting infants gut could be predicted from their mothers. The AUC values averaged across the six studies were proven to be higher than 0.5, from 0.53 for *P. distasonis* and 0.75 for *B. longum* (Figure 6b). Given the influences of the mode of delivery on the microbial composition in infants, we stratified the samples based on the mode of delivery and retrained the random forest classifier separately with LOSO approach. The AUC scores obtained from mothers giving birth vaginally were comparable to the pooled samples, while the predictive performances with the gut microbiota of mothers delivering via C-section decreased (Wilcoxon test, *p* < .01; Figure 6c).

In order to evaluate the number of microbial features that were necessary to obtain the predictive performance values comparable to those achieved using the full set of features, the top-ranking features were chosen based on the mean decrease in GINI from the previous random forest classifier with LOSO approach, and then retrained for a new random forest classifier for each species to compute AUC values. We found that the stable AUC values averaging over 26 species were achieved using as few as 10 species in most cases (Figure 6e), emphasizing the high predictive power of the top features. We then tried to explore the profile and exact contribution of top features in the model for each shared species. The results indicated that the predictive species with the highest rank of mean decrease in GINI corresponding to each random forest classifier was the maternal gut species for which the classifier was trained in cases of 21 species, such as *B. longum* (Figure 6d), except for *B. ovatus, B. uniformis, F. prausnitzii, R. torques*, and *Subdoligranulum* spp.

## Discussion

Direct comparisons of the infant gut microbiota to their matched mother have been limited by the taxonomic resolution provided by 16S rRNA gene profiling,^30^ small sample sizes from a few to tens at higher taxonomic resolution^8,15,19,20^ and overlooked aspects in microbial metabolic functions. In the present study, we applied a meta-analysis approach to analyze whole metagenomic shotgun sequencing data with 376 matched mother–infant dyads from eight studies across six countries. This has enabled us to identify and characterize global distinct and identical features of the mother–infant gut microbiota across cohorts at species, strain, and functional levels. Despite differences in various clinical covariates (e.g., maternal BMI, antibiotic interventions for mothers and infants, mode of delivery), we identified a core set of shared species across cohorts. Compared to non-shared species, the shared species had similar strain heterogeneity but higher functional capacities, suggesting a critical role for the shared species in early colonization. We found the biosynthesis of purine nucleotides, proteinogenic amino acids, and folate were particularly enriched in infants compared to their mothers. In addition, prediction of occurrences of shared species and strains in infants based on their maternal gut microbiota using different machine learning approaches was proven to be moderately accurate.

Beyond the striking differences between mothers and infants at three months of age,^8^ we found that these differences diminished significantly as the infant grew until 1 year of age in terms of both microbial diversity and composition. Increases in the relative abundances of *Bacteroides* (*B. ovatus* and *B. uniformis), Eubacterium* (*Eubacterium rectale*), and *Faecalibacterium* (*F. prausnitzii*) and decreases in *Bifidobacterium* (*B. longum, B. pseudocatenulatum*, and *B. bifidum*) and *Escherichia* (*E. coli*) occurred from approximately 6 to 12 months, which may be due to a shift from exclusive breastfeeding to partial and/or complete solid food.^6,10,31^ However, these findings were based on the relative abundance but the absolute changes in these species still remain unclear. Individual biomarker discovery could be sensitive to the variability between studies and the microbial shifts in the host itself, due in part to diet and host health status. Across studies, we have identified a set of 26 mother–infant shared species (species of *B. uniformis, B. vulgatus, B. longum, P. distasonis, P. merdae* confirmed at strain level) with high prevalence and relative abundance. When taking delivery mode into account, the number of mother–infant shared species in C-section-born infants was much less than those born vaginally, reflecting the various origins of microbiota from C-section-born infants, e.g., the maternal skin and hospital environment.^32,33^ Considering the potential contribution of other microbial sources (e.g., vagina, oral cavity, and breast milk) to infant gut microbiota, we cannot quantify the extent of the contribution of maternal gut microbiota in the current study. For example, vaginal microbiota has been frequently reported to be transmitted to infant gut microbiota, which is typical for those born vaginally.^17,34,35^

We took the longitudinal nature of included studies to observe that the infant gut strains showed higher stability within the majority of specific species than that of mothers sampling over pregnancy and lactation. This result indicated the high chance of strain shifts in mothers, expanding the existing evidence that the gut microbial profile dramatically changed during pregnancy.^36,37^ However, the strains of *B. longum* as the earlier and dominant colonizer in infant gut showed higher variability across different time points than those residing in mothers. It is well known that members of  the *B. longum* species are among the main utilizers of human milk oligosaccharides (HMO) due to the presence of specific genes in their genomes.^12,38^ However, the majority of *B. longum* strains that were found to contain HMO utilization genes during breastfeeding no longer carried these genes after the cessation of breast milk.^3^ Our results together with the previous observations corroborate the hypothesis of the strain-specific functional adaptation in the gut microbiome during early life. In addition, we found that strains of *R. torques* exhibited the highest diversity longitudinally in both mothers and infants, which has not been reported previously. *R. torques* appeared to be associated with breastfeeding in infants^11^ and some disorders, such as autism spectrum disorder^39^ and amyotrophic lateral sclerosis.^40^ These observations indicate that strains of *R. torques* are susceptible to extrinsic factors, which is further confirmed by our results at strain level in both infants and adults. However, the mechanism of strain shifts of *R. torques* in different environmental niches remains to be determined.

Confirmation of the microbial mother-to-infant vertical transmission at higher taxonomic resolution is desirable but still faces challenges.^41^ We used a single-nucleotide variant strain profiling approach on the marker genes of each species^21,24^ and corroborated evidence of microbial mother-to-infant vertical transmission events at strain level in a total of 103 species, which enlarge the number from individual studies with limited sample size.^8,17^ The number of shared strains increased as infants aged in the first year of life. In addition, we confirmed that the transmission frequency of *Bifidobacterium* spp., *Bacteroides* spp., and *E. coli* was much higher in the first year of life, in line with previous studies.^19,42^ Although mother’s gut-sourced strains are likely to be more ecologically adaptable in infants,^17^ our results indicate that the same strains from other sources also showed comparable stability and fitness compared to those from mothers’ gut. This suggests that early microbial acquisition is controlled, rather than occurring by chance. Although it is not possible with our current data sets to exactly specify the “other sources”, this still highlights the need to isolate, identify, and characterize the potential species out of the scope of the human gut. In terms of specific functional traits, the transmitted strains contributed more various functions in infants than those from other sources. Meanwhile, the clinical significance of different microbial metabolic functions based on different origins, as we observed from the shared and non-shared strains, remains to be determined.

The biosynthesis of purine nucleotides, proteinogenic amino acids, and folate was more active in infants; while the abundance of starch degradation and phospholipid biosynthesis was higher in mothers. These differences truly reflect the distinction in functional capacities of the gut microbiota between mothers and infants, which is likely attributed to microbial adaptation to different dietary composition and intake at different stages of life.^43^ However, higher similarity of microbial metabolic pathways compared to taxonomic profiles was observed, which was observed among unrelated subjects,^26^ and Human Microbiome Project.^44^ This may be attributed to the presence of “core” microbial community functions that are essential for the whole microbial tree of life and adapted to the specific ecological niches.^3^

In addition to providing further evidence that maternal characteristics (including BMI before pregnancy and antibiotic intervention during delivery) and mode of delivery are critical factors affecting the developmental trajectory of the gut microbiota in the first year of life,^45,46^ our meta-analysis has highlighted how the mode of delivery affects the degree of microbial mother–infant shared species and the number of vertically transmitted strains, which is still unknown. We found that C-section birth decreased the abundance of shared species from *Bacteroides, Bifidobacterium*, and *Parabacteroides*; while shared species commonly associated with preterm birth (e.g., *H. parainfluenzae, R. mucilaginosa*, and *Veillonella* spp.) increased^47,48^ in the first year of life. At strain level, the mother-to-infant microbial transmission was longitudinally more prevalent in vaginally born neonates. Although the potential association of the microbiota with the increased risk of adverse health outcomes in C-section-born infants^49,50^ due to the impact of early microbial colonizers on the immune system^51,52^ may exist, the causality between these altered microbiota and adverse health outcomes remains to be identified. In addition, whether those mother–infant shared species associated with C-section have implications for the intergenerational transmission of chronic diseases that have been related to gut microbiota dysbiosis is warranted. Furthermore, our results showed that this disruption could be alleviated partially by exclusive breastfeeding postpartum.

Prediction of host health status based on gut microbiota has been conducted for some diseases, such as asthma^53^ and colorectal cancer.^54,55^ However, to date, prediction of the gut microbiota in infants has not been addressed, which, if possible, may guide us to design specific probiotic supplementation for mothers and infants. Given maternal microbiota as one major source for the infant gut microbiota, we firstly attempted to develop machine learning models with different algorithms to predict occurrences of the core set of 26 mother–infant shared species in infants that were identified in the current study based on their maternal gut microbiota. This resulted in moderate performance accuracy with an average AUC value of 0.69 with random forest for the core set of shared species across studies. Importantly, the highly similar performances were achieved with another machine learning approach (average AUC value 0.72 for gradient boosting). It was also apparent that these models generated with LOSO approach were not heavily biased among studies, although the sample size of each study varied. The microbial mother-to-infant vertical transmission was also proven to be predicted to some extent using the same way as shared species. In addition, we found that the main contributor in the majority of random forest classifiers was the species shared by mothers and infants, indicating the high chance of the existing maternal species to be shared in their infants, irrespective of the abundance. Probiotic supplementation for the mother-to-be has been used to prevent and manipulate the microbiota dysbiosis during pregnancy,^56,57^ as well as for infants during early life, particularly for C-section-born infants who lack members of *Bacteroides* in their gut microbiota during first few months.^19,31^ Our findings would be useful for future probiotic intervention studies and next-generation probiotic development, and underscore the possibility to predict occurrences of shared species and strains in early life based on maternal gut microbiota.

Taken together, our meta-analysis is a combined analysis of all the available metagenomic sequencing data sets with the collection of mother–infant dyads samples, and the results uncovered the global distinct and identical microbial signatures from species, strains to metabolic functional levels. The findings could form a basis of future mother–infant gut microbiota studies. The core set of shared species across studies may potentially be explored as next-generation probiotics for infants, in particular those born by C-section, and their ecological roles in the acquisition and development of microbiota in early life need to be further addressed with large-scale, long-term cohort studies. The clinical implication of differences between shared and non-shared species on health outcomes of infants remains to be fully understood.

## Materials and methods

### Study selection

PubMed with the terms “(maternal or mother) AND (infant) AND (microbiome or microbiota) AND (metagenomic or metagenomics)” was utilized to search studies that included fecal metagenomic data from mothers and paired infants (up to 13.12.2019). Studies that collected and sequenced fecal samples by shotgun metagenomics from infants and their matched mothers were included in this meta-analysis with the non-stool samples removed. The justification of studies to be excluded or included is summarized in Supplementary Table S1. Of the eight studies that fulfilled the criteria for the meta-analysis, two each were from Italy and Finland, and one each from Sweden, the United States, Luxembourg, and the United Kingdom. A summary of the metadata of included studies is presented in Supplementary Table S1.

## Data retrieval and processing

The raw FASTQ files were downloaded directly based on the published accession number. The downloaded raw FASTQ data of each study were reprocessed separately with consistent processing to avoid the biases from bioinformatics analyses. The metagenomic shotgun sequencing data were trimmed and human reads (hg19 human reference genome) were filtered by using KneadData (v0.7.2) with the default parameters. Quality controlled data were taxonomically profiled at the species level with relative abundance by MetaPhlAn2 (v2.7.5)^58^ using the default settings, which uses unique clade-specific marker genes identified from ~17,000 reference genomes to provide pan-microbial quantification at the species level, including bacteria, archaea, microbial eukaryotes, and viruses.

## Mother–infant shared microbiota at microbial species and strain level

The species that were shared between infants and their mothers were determined based on the presence/absence in the mother–infant pair regardless of their relative abundance. Identification of shared microbial strains for species detected by MetaPhlAn2 was performed by a SNP calling method.^21^ The quality controlled metagenomic shotgun sequencing data were subjected to StrainPhlAn with the option “–marker_in_clade 0.2, – sample_in_marker 0.09, and – gap_in_sample 0.5” as previously described,^20^ generating one dominant strain for each species. Afterward, a species-specific phylogenetic tree for each strain was generated by using RAxML.^59^ Strain distance for any pairwise mother–infant samples was defined as the normalized pairwise phylogenetic distance on the corresponding tree. The strains were considered to be shared by infants with their mothers if the strain distance of mother–infant dyads was less than the conservative threshold of 0.1, otherwise, the strain was identified to be distinct.^17,19^ The marker gene-based SNP haplotype^24^ of the dominant strain per species was obtained from StrainPhlAn.

## Mother–infant shared metabolic functional profiling

Functional metagenome annotation including pathway and gene-family abundances was conducted by HUMAnN2 v0.11.2,^60^ which constructs a sample-specific reference database by concatenating precomputed, functionally annotated pangenomes of species in the sample detected by MetaPhlAn2 for each metagenome. After mapping metagenomic reads against this database to quantify gene presence and abundance in a species-stratified manner with the unmapped reads that were further mapped against UniRef90 by a translated search, HUMAnN2 reconstructed the profile of metabolic pathways based on the gene families outputs annotated to MetaCyc reactions. Pathway abundance files from each sample were then joined into one abundance table with “humann2_join_tables”. The combined pathway tables for each study were normalized to relative abundance with using “humann2_renorm_table”. Gut metabolic functions were summarized across metabolic pathways based on the parent class assigned to each pathway.

## The random forest-based machine learning approach

The taxonomic species-level relative abundances in mothers obtained from MetaPhlAn2 were used for machine learning analysis to predict occurrences of shared species in their infants from the subset of six studies (Bäckhed et al.,^9^ Chu et al.,^16^ Ferretti et al.,^17^ Shao et al.,^19^ Wampach et al.,^20^ and Yassour et al.^8^). The LOSO approach was applied consisting of training the model on the pooled samples from all studies except the one used for model testing. To ensure the presence of shared and non-shared species in each study thus allowing the prediction and evaluation of the model, the core set of 26 shared species that contained two patterns among the samples in each of the six studies were predicted for their occurrences in infants. The initial features for prediction were firstly pre-processed using the nearZeroVar function with default settings to remove the features with zero-variance or near-zero-variance. The highly correlated features were further filtered with the findCorrelation function from R package “caret”.^61^ As a result, a total of 175 predictive features out of 569 in the overall maternal species were retained to train random forest classifiers. The randomForest function from R package “randomForest”^62^ was carried out to train the model with 1000 estimator trees and “mtry” was left as the default setting. Predictions and performance metrics were calculated using the predict, and prediction, performance functions from R package “ROCR”.^63^ Predictions of strains whether they were transmitted from mothers to infants were conducted in the same way as described above for mother–infant shared species prediction with LOSO approach. There were five strains to be retained as they were observed with transmitted and non-transmitted patterns in each of the six studies.

## The stochastic gradient-boosting (GBM) machine learning approach

In parallel with random forest analysis, the retained features were further analyzed with GBM machine learning with R package “gbm”.^64^ which builds one tree at a time where a new tree helps to correct errors made by the previously trained tree and combines results along the way while random forest trains each tree independently and combines results at the end of the process. The LOSO approach was applied with the GBM parameters (i.e., interaction depth 1; number of tree 100, shrinkage 0.1; the number of minimum observation in the terminal nodes of the trees 5; number of cross-validation folds 5). The AUC was generated using the roc function from R package “pROC”.^65^

### Statistical analysis

All statistical analyses were carried out in the R software environment (v3.6.1) using the appropriate functions. For comparisons between the abundances of microbial taxa and functional profiles, the non-parametric test with two-sided Wilcoxon rank-sum test (Wilcoxon test) for two groups and Kruskal–Wallis test for more than two groups were employed, unless otherwise specified. Ordination was performed using PCoA based on Bray–Curtis dissimilarity. The significance value was determined with PERMANOVA implemented through the function adonis in the “vegan”^66^ package based on 1000 permutations. Bray–Curtis distance was also measured to compare mother and infant samples belonging to the same family and unrelated mother–infant pairs.

To quantify the dynamic changes of the feature of the gut microbiota, we stratified the continuous sampling time points of mothers into three categories (pregnancy, delivery, and postpartum) and infants into six categories (birth, one week, one month, three months, six months, and twelve months) when necessary. The three categories for maternal samples were: pregnancy (189–224 gestational days; *n* = 53, mean of days (MD) = 199.6, 95% CI = [195.1; 204.0]), delivery (0–7 days, *n* = 338, MD = 0.56, 95% CI = [0.41; 0.71]), postpartum (8–480 days, *n* = 76, MD = 69.4, 95% CI = [53.4; 85.3]). The six categories for samples from infants included: birth (0–1 days, *n* = 71, MD = 0.51, 95% CI = [0.39; 0.63], referred to as birth), one week (2–7 days, *n* = 378, MD = 5.25, 95% CI = [5.07; 5.43], referred to as 1 wk.), one month (8–30 days, *n* = 210, MD = 21.0, 95% CI = [20.1; 22.0], referred to as 1 mo.), 2–3 months (31–90 days, *n* = 75, MD = 72.1, 95% CI = [68.0; 76.1], referred to as 3 mo.), 4–6 months (91–180 days, *n* = 127, MD = 130.6, 95% CI = [126.9; 134.3], referred to as 6 mo.), and seven to twelve months (181–480 days, *n* = 157, MD = 332.7, 95% CI = [323.3; 342.1], referred to as 12 mo.).

The significance of differential abundance of per phylum/genus/species or metabolic functions/pathways between mothers and infants was examined using a blocked (by “study”) Wilcoxon test implemented in the R package “coin”.^67^ and the produced *p*-values were corrected for multiple testing using the Benjamini–Hochberg FDR (of 5%, FDR).

The proportion of explained variance (*R*^2^) and significance of each clinical covariate in terms of the composition of shared species by mothers and infants were stratified by “sampling time points” (as described previously) and then quantified by a cross-sectional and univariate PERMANOVA calculated based on Bray–Curtis dissimilarity between samples for each covariate, as implemented in the adonis function from R package “vegan”.^66^ Samples with missing metadata for the given covariates were removed before running each cross-sectional PERMANOVA, and the covariate consisting of any group with sample size less than three was excluded. Effect size and statistical significance were determined by 1000 permutations, and *p*-values were adjusted with Benjamini–Hochberg FDR method for multiple hypothesis testing. The covariates included mode of delivery (vaginal vs. C-section), studies, feeding regimen (exclusive vs. non-exclusive breastfeeding), postnatal antibiotic intervention in infants (true vs. false), gestational age (full-term vs. preterm), maternal age (19–29; 30–34; 35–39; 40–50), maternal antibiotics before delivery (true vs. false), maternal intrapartum antibiotics (true vs. false), birth weight (low birth weight: <2500 g; normal birth weight: 2500–4000 g; high birth weight: > 4000 g), C-section type (elective vs. emergency), maternal BMI before pregnancy (normal: 18.5 ≤ BMI < 25; overweight/obese: BMI ≥ 25) and gender (female vs. male) on the mother–infant shared microbiota.

Associations between each microbial taxonomic and functional feature abundance and a specific covariate (i.e., mode of delivery, feeding regimen, and maternal characteristics) were quantified using MaAsLin^68^ while accounting for the other potential covariates. Boosted additive general linear models using a variance-stabilizing arcsin square root transform on relative abundances were then used to determine the significance of putative associations. All 12 covariates tested in the PERMANOVA for variance contribution were included in the adjustment, along with the infant age and sequencing depth and used as fixed effects. The default MaAsLin parameters were applied providing both a nominal *p*-value and a Benjamini–Hochberg FDR-adjusted *p*-value (FDR value). Here, microbial features with FDR < 0.25 (default value)^3,11,19^ were reported.

## Supplementary Material

Supplemental Material

## Data Availability

All metagenomic sequencing data sets analyzed in this study are available from public sources. The data supporting the findings of this study are available within the paper and additional files. The analysis software packages used in this study for quality control, taxonomic, and functional profiling are publicly available in bioBakery at https://bitbucket.org/biobakery/biobakery/wiki/Home, which have been referenced as appropriate.
