# Supplementary material for: Metagenomic analysis of mother-infant gut microbiome reveals global distinct and shared microbial signatures
Source: Gut Microbes. 2021 May 7;13(1):1911571. doi: 10.1080/19490976.2021.1911571 (PMC8115609; doi:10.1080/19490976.2021.1911571)
Supplement: Supplemental Material [file KGMI_A_1911571_SM8722.zip › Supplementary information/Supplemental_figures_and_table_legends_corrected.pdf]

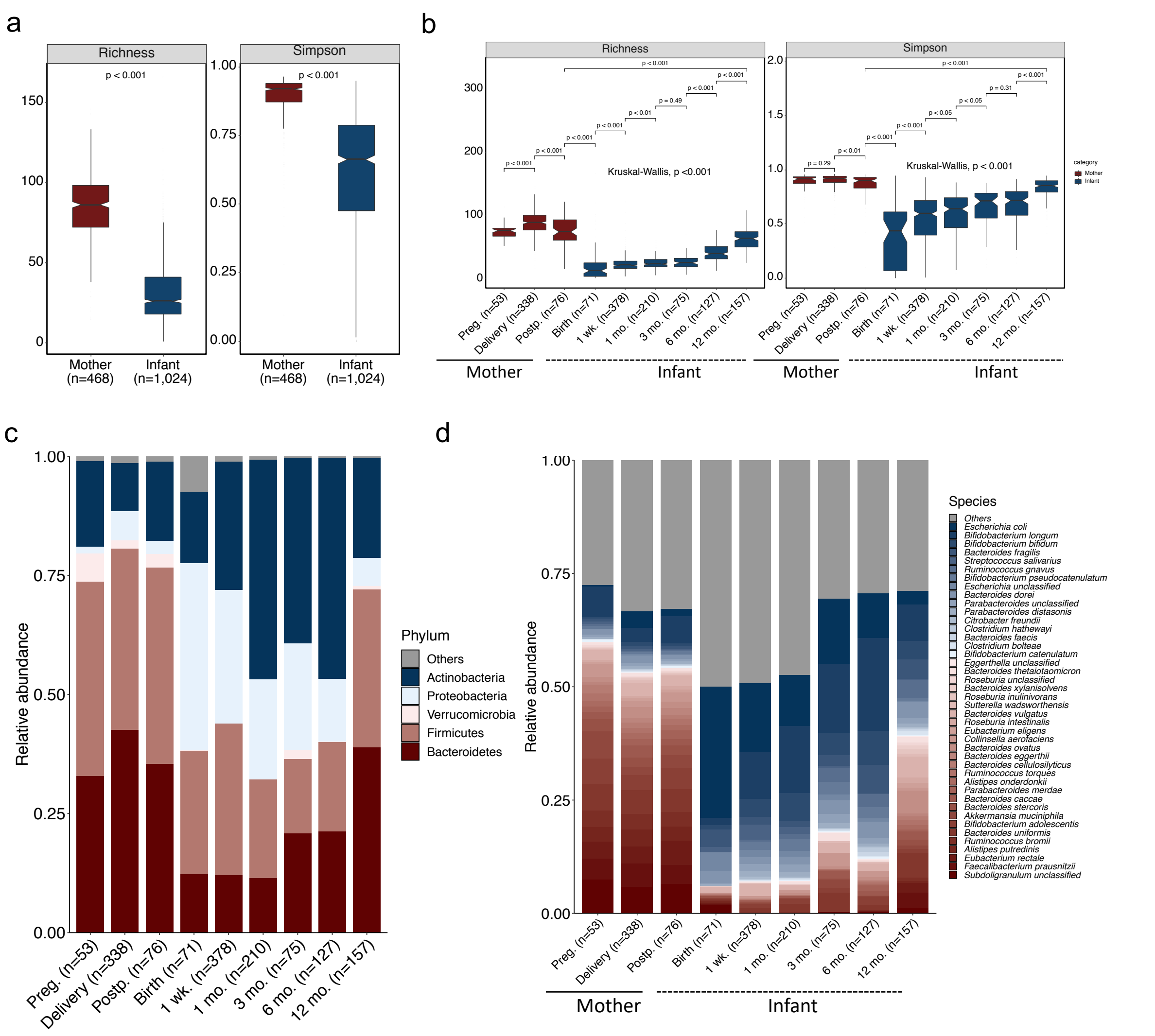

Figure S1. Comparisons of gut microbiota diversity and composition of mother-infant dyads. (a) Species richness and alpha diversity (Simpson diversity index) of mothers (n = 468) and infants (n = 1,024). The p-value was computed using a blocked (by "study") Wilcoxon test from R package "coin". (**b)** Species richness and Simpson diversity index of mothers and infants stratified by "sampling time points" into three categories for mothers and six categories for infants. The p-values were computed using Wilcoxon test. The overall p-value (on top) was calculated with a blocked (by "study") Kruskal-Wallis test from R package "coin". (**c)** Comparisons of the mean relative abundance of phyla from stools of mothers and infants. Only the phyla that differed (FDR < 0.05, Wilcoxon test blocked by "study") among mothers and infants, with > 1% mean relative abundance in either maternal or infant samples across all "sampling time points", are plotted. (**d**) Dynamic changes of the species that differed (FDR < 0.05, Wilcoxon test blocked by "study") between mothers and infants, with > 0.1%

mean relative abundance and at least 5% prevalence among maternal and infant samples across all "sampling time points", respectively. The mean relative abundances of phyla and species in blue are higher in stools of infants, and those in red are higher in mothers.

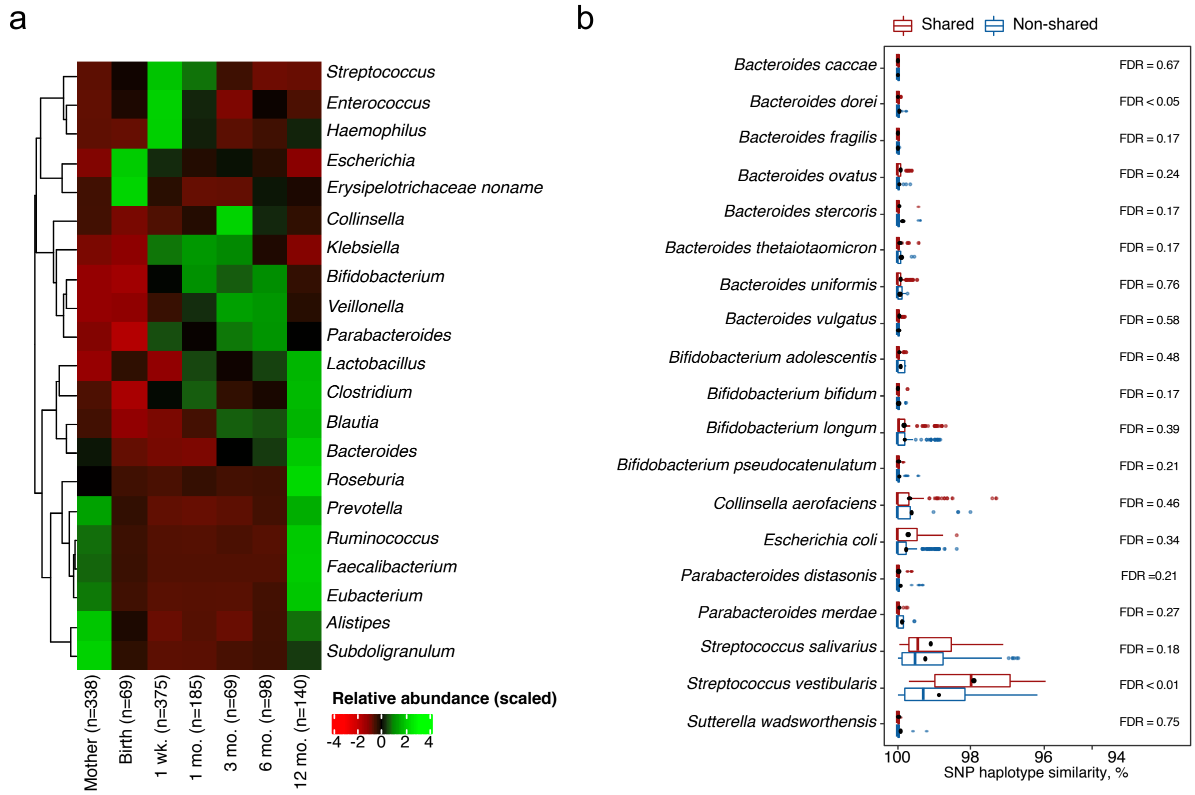

Figure S2. Mother-infant shared genera and SNP haplotype similarity comparison between shared and non-shared species. (a) Longitudinal changes in the mean relative abundance of genera shared by mothers and infants, with > 0.3% mean relative abundance and at least 5% prevalence of infant samples across all the "sampling time points". (**b)** SNP haplotype similarity of each species based on all pairwise comparisons (dominant strain per species) of the marker genes, and stratified to shared-species and non-shared species. Species containing at least 10 comparisons in both strata are shown. The significance (p-values adjusted by Benjamini–Hochberg FDR method on the right of the bars) of the difference in similarity between shared and non-shared species was determined by Wilcoxon test. The solid black point indicates the mean of strain similarity.

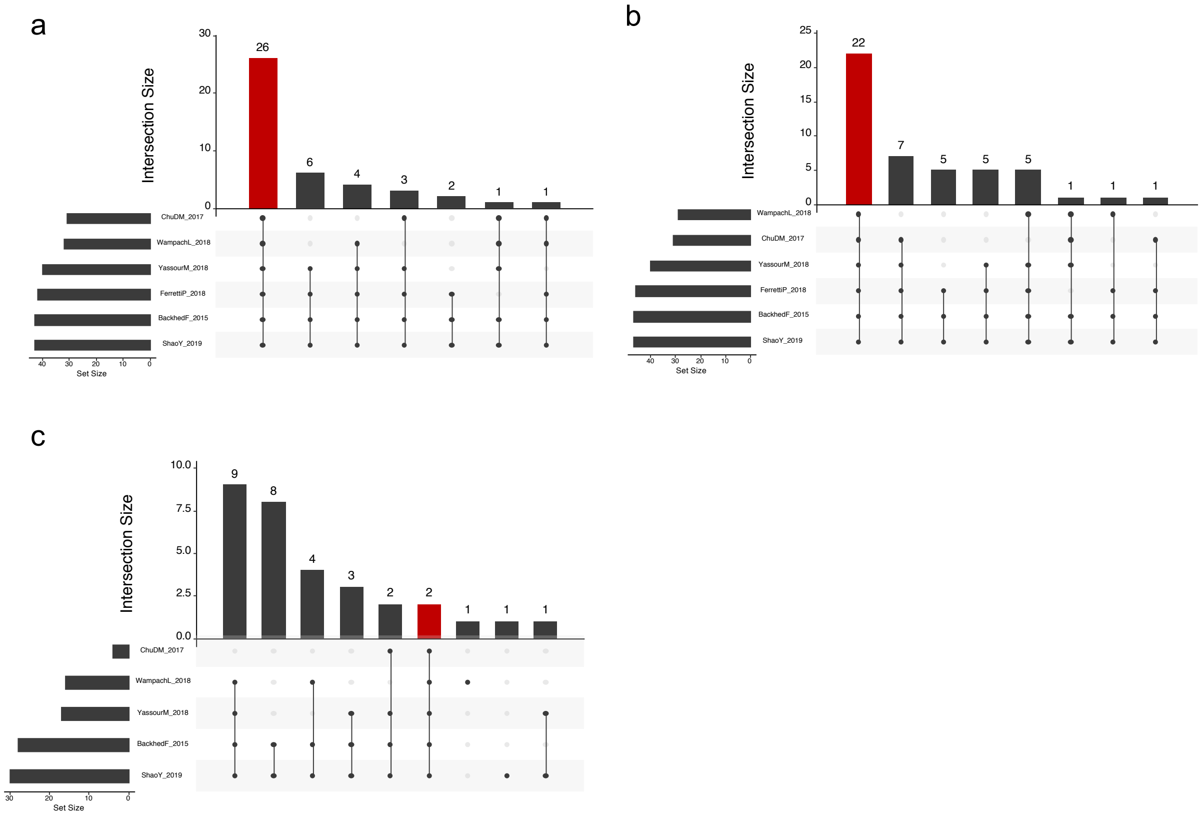

Figure S3. UpSet plot illustrating the mother-infant shared species across studies. (a) All infants including born vaginally and by Cesarean section in a subset of six studies. **(b)** Infants born vaginally available in six studies. (**c**) Infants born by Cesarean section available from a subset of five studies. The vertical bars and the number indicated on the top indicate the mother-infant shared species observed in corresponding studies with black dots in the lower panel. Horizontal bars in the lower panel indicate the total number of mother-infant shared species in each study. The red bar in each figure indicated that the mother-infant shared species were found in all studies. Only species with > 0.1% mean relative abundance and at least 5% prevalence across all infant samples were analyzed.

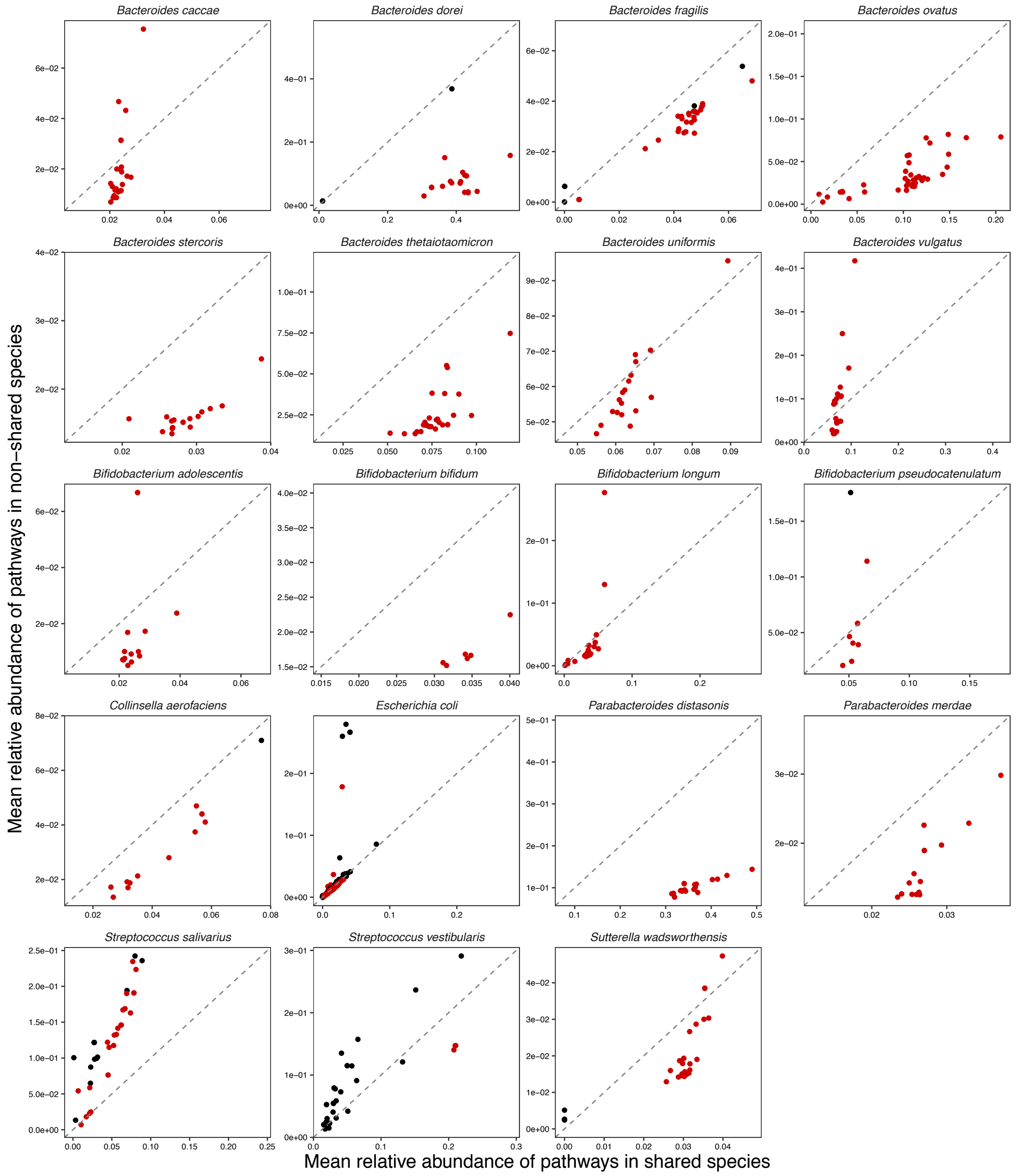

Figure S4. Comparison of metabolic pathways in microbial strains present in infants that are shared or not shared with mothers. The pathways colored in red circles below the diagonal line were considered to be significantly higher (FDR < 0.05, Wilcoxon test) in the mean relative abundance in shared stains than non-shared stains, or *vice versa*.

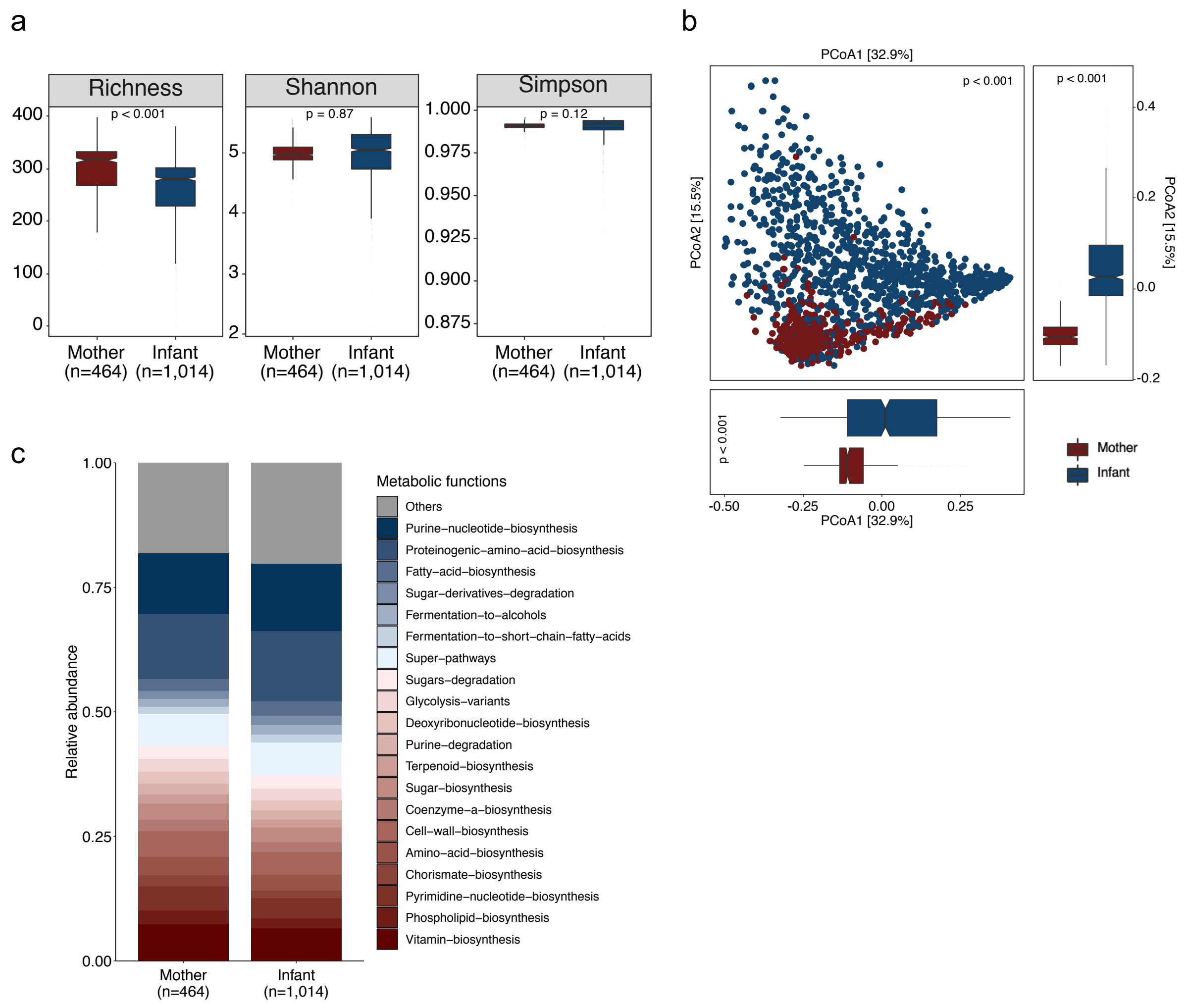

Figure S5. Comparison between mothers and infants in terms of diversity and composition of gut microbial metabolic functions. (a) Microbial metabolic pathway richness and alpha diversity (Shannon diversity index and Simpson diversity index) of mothers and infants. The p-value was computed using a blocked (by "study") Wilcoxon test. **(b)** Principal coordinate analysis (PCoA) of samples of mothers and infants from all the eight included studies based on Bray-Curtis dissimilarity of microbial metabolic pathway. The boxplots on the right side and below show samples of mothers and infants projected onto the first two principal coordinates, respectively. The p-values were calculated by the adonis (permutations = 1,000) function from the R package "vegan" for the PCoA plot, and by a blocked (by "study") Wilcoxon test from R package "coin" for the boxplots. **(c)** Comparison of the mean relative abundance of metabolic microbial functions from mothers and infants. Only the functions that differed (FDR < 0.05, Wilcoxon test blocked by "study") among mothers and infants, with > 1% mean relative abundance and at least 5% prevalence among maternal

and infant samples, respectively, are plotted. The mean relative abundances of metabolic functions in blue are higher in infants, and these in red are higher in mothers.

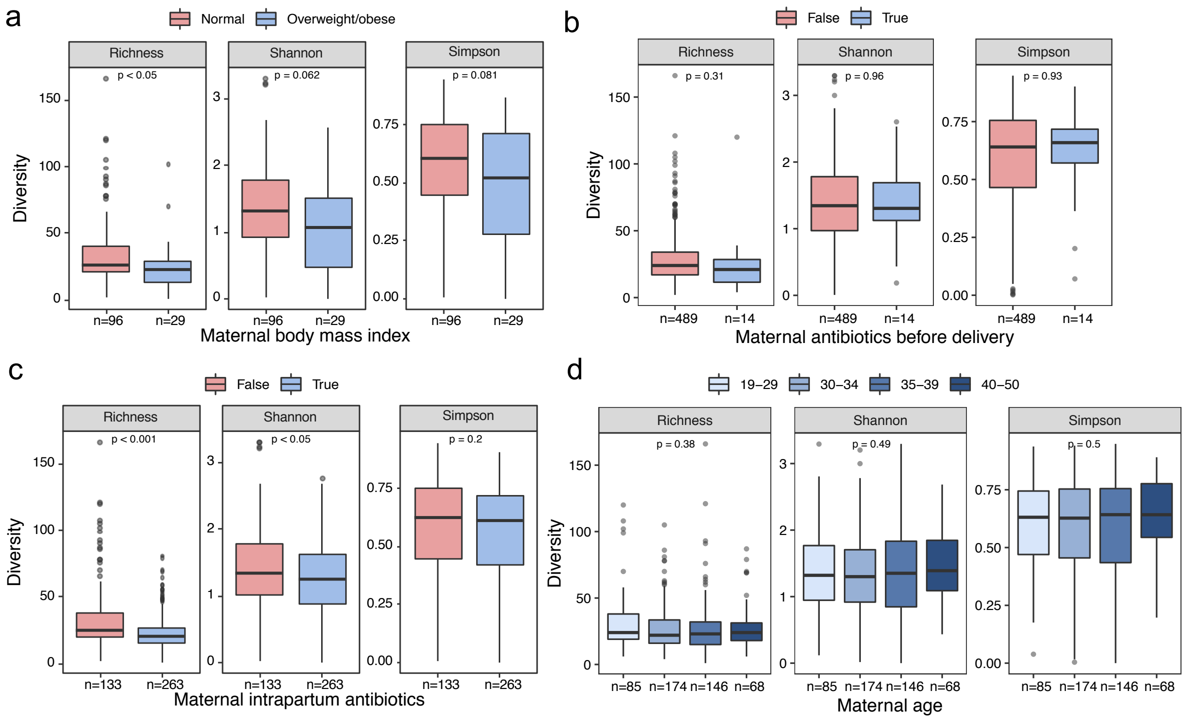

Figure S6. Influence of maternal characteristics on the alpha diversity of infant gut microbiota at the species level. (a) Maternal body mass index before pregnancy (b) Maternal antibiotics before delivery (c) Maternal intrapartum antibiotics (d) Maternal age. The p-values were computed with Wilcoxon test.

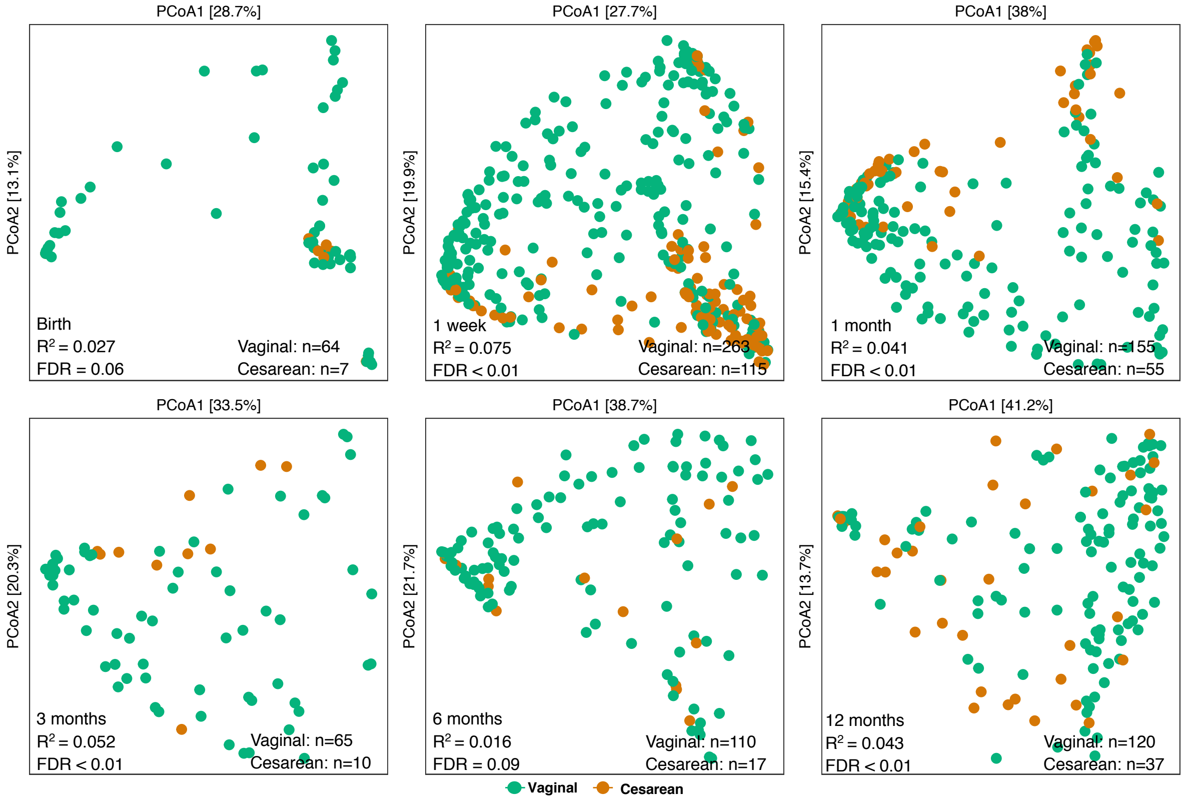

Figure S7. Principal coordinate analysis (PCoA) of infants samples per timepoint stratified according to mode of delivery based on Bray-Curtis dissimilarity at the genus level. The microbial variation (R^2) explained by the mode of delivery was calculated by the cross-sectional PERMAOVA as implemented in the adonis function from R package “vegan”. The p-values were adjusted with Benjamini–Hochberg FDR method for multiple hypothesis testing.

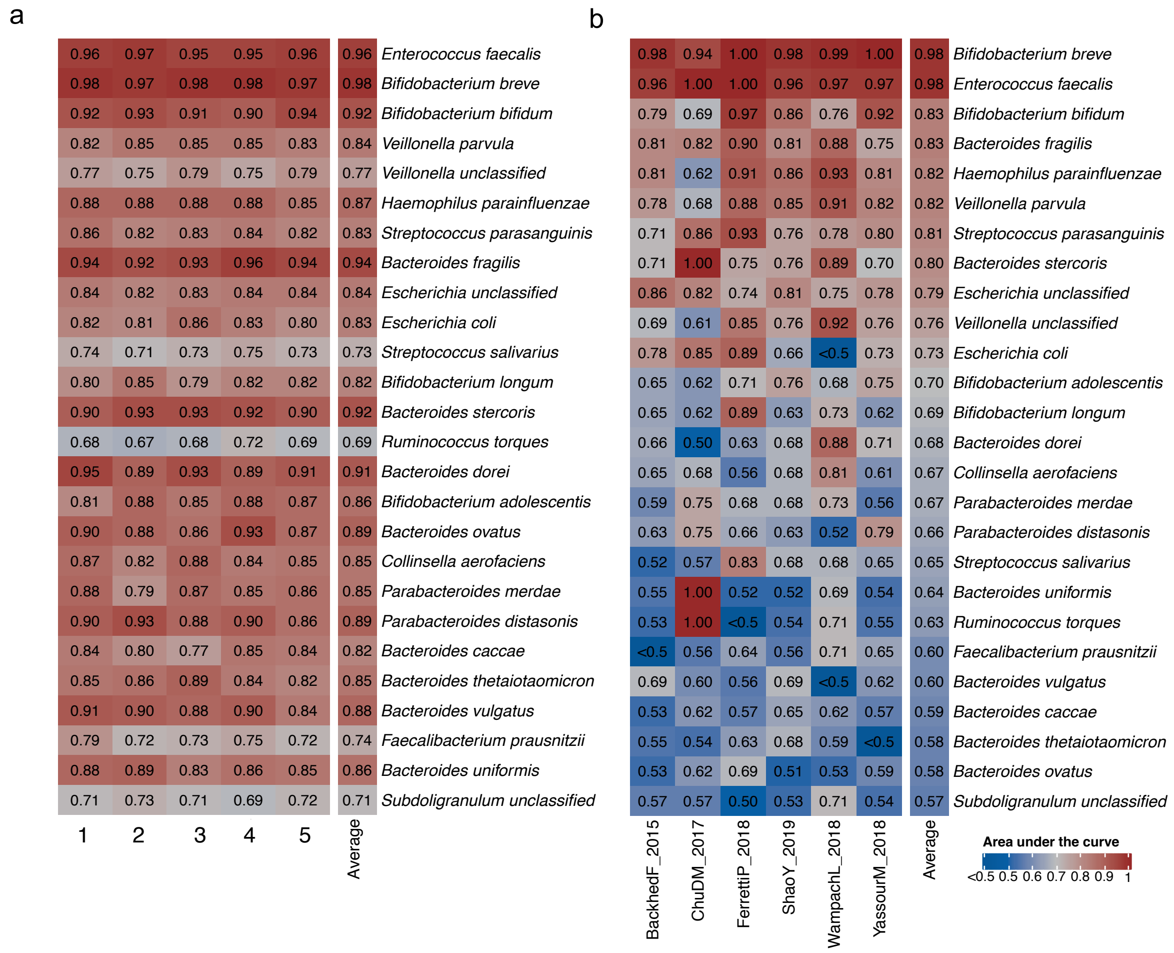

Figure S8. Assessment of the prediction performance of the mother-infant shared species. (a) The area under the curve (AUC) matrix obtained from random forest model with for the core set of 26 species when the pooled samples from six studies were randomly separated into a training set and validation set (70% and 30% respectively) with repetition for five times. Each column refers to the performance of machine learning in each repetition. **(b)** The AUC matrix obtained with stochastic gradient-boosting (GBM) machine learning approach and leave-one-study-out (LOSO) approach for the core set of 26 mother-infant shared species across six studies. Each column refers to the performance of machine learning by taking all but the dataset of the corresponding column and applying it to the dataset of the corresponding column.

Supplemental table legends

Table S1. PubMed results of the meta-analysis search and reasons for inclusion/exclusion of studies, and the metadata of each included study, as well as the summarized metadata.

Table S2. Relative abundance averaged across "sampling time points" of phylum, genus, and species with significantly differences (FDR < 0.05, Wilcoxon test blocked by "study") between maternal and infant stools.

Table S3. Dynamic changes over the first year of life in relative abundance of phylum, genus, and species.

Table S4. Relative abundance of mother-infant shared species.

Table S5. Dynamic prevalence of mother-infant shared strains stratified by "sampling time points".

Table S6. Significantly different metabolic pathways and functions between mothers and infants.

Table S7. Mother-infant shared microbial metabolic pathways with significantly differences between mothers and infants in at least one time point.

Table S8. Influence of maternal characteristics on the infant gut microbial genera and species analyzed by MaAsLin.

Table S9. Significantly different gut genera and species of vaginally- and Cesarean section-born infants analyzed by MaAsLin.

Table S10. Dynamic prevalence of mother-infant shared strains stratified by mode of delivery and "sampling time points".

Table S11. Influence of Cesarean section and breastfeeding on mother-infant shared genera and species analyzed by MaAsLin.
